# Interactions of Aging, Overload, and Creatine Supplementation in Rat Plantaris Muscle

**DOI:** 10.4061/2011/393416

**Published:** 2011-08-11

**Authors:** Mark D. Schuenke, Naomi E. Brooks, Robert S. Hikida

**Affiliations:** ^1^Department of Anatomy, College of Osteopathic Medicine, University of New England, Biddeford, ME 04005, USA; ^2^Department of Physiological Sciences, Stellenbosch University, Stellenbosch 7602, South Africa; ^3^Ohio Musculoskeletal and Neurological Institute (OMNI), Department of Biomedical Science, Ohio University College of Osteopathic Medicine, Athens, OH 45701, USA

## Abstract

Attenuation of age-related sarcopenia by creatine supplementation has been equivocal. In this study, plantaris muscles of young (Y; 5m) and aging (A; 24m) Fisher 344 rats underwent four weeks of either control (C), creatine supplementation (Cr), surgical overload (O), or overload plus creatine (OCr). Creatine alone had no effect on muscle fiber cross-sectional area (CSA) or heat shock protein (HSP70) and increased myonuclear domain (MND) only in young rats. Overload increased CSA and HSP70 content in I and IIA fibers, regardless of age, and MND in IIA fibers of YO rats. CSA and MND increased in all fast fibers of YOCr, and CSA increased in I and IIA fibers of AOCr. OCR did not alter HSP70, regardless of age. MND did not change in aging rats, regardless of treatment. These data indicate creatine alone had no significant effect. Creatine with overload produced no additional hypertrophy relative to overload alone and attenuated overload-induced HSP70 expression.

## 1. Introduction 

Aging is associated with reductions in skeletal muscle mass and strength (sarcopenia), which may lead to impaired mobility in older individuals. Despite a litany of research on sarcopenia, the underlying mechanisms remain to be elucidated. Comprehensive reviews of the potential mechanisms behind sarcopenia are available (e.g., Ryall et al. 2008 [1]). In brief, declines in muscle-specific stem cells (satellite cells); increases in myostatin, a negative regulator of muscle growth; changes in circulating hormone levels; impairment of neuromuscular function; increased myonuclear apoptosis have all been proposed as mechanisms underlying sarcopenia. 

Creatine (C_4_H_9_N_3_O_2_) supplementation is a popular dietary supplement with recreational and professional athletes. It has been shown to increase exercise performance [2, 3] and strength [4]. Creatine is a naturally occurring molecule in skeletal muscle and can be synthesized or obtained in the diet. Creatine and phosphocreatine (PCr) are involved in coupling anaerobic metabolism with ATP demand [5]. Increased intramuscular levels of PCr may permit increased temporal buffering of ATP during high-intensity muscle contraction, and increased creatine levels may allow increased PCr resynthesis [6]. In so doing, creatine supplementation may indirectly increase muscle mass and strength by allowing training at higher workloads [7]. However, the mechanisms behind the enhancement of performance and increases in muscle strength by creatine supplementation have not been fully elucidated. 

The use of a dietary supplement, such as creatine, to counteract sarcopenia is not unprecedented. Other nutritional interventions have produced positive results against sarcopenia. High protein diets may be beneficial for stimulating protein synthesis in the muscles of aging individuals [8]. The use of creatine in elderly populations is of interest because of its potential to increase energy production, fat-free mass, and muscle mass [9]. Combined, these effects would increase the ability of the elderly to perform everyday tasks such as rising from a seated position and walking. However, older individuals appear to respond differently than young individuals to creatine supplementation [10]. Short durations of creatine supplementation alone in elders have shown mixed results. Creatine supplementation for five days in aging population had no effect on isometric strength [11, 12]. Similarly, five days of creatine supplementation had no effect on pedaling performance in cycling-trained elderly subjects [13]. Conversely, 5 days of creatine supplementation increased anaerobic power and work capacity of sedentary elderly subjects [13]. Short-term creatine supplementation has also produced increases in strength and fat-free mass of elderly men [14] and women [15]. 

Longer-term creatine supplementation combined with resistance training in the elderly has also demonstrated varied responses. The combined treatments resulted in anything from no extra benefit after 8 weeks of strength training and supplementation [16], to small increases in muscle strength after 12 weeks [17], to moderate increases in strength and creatine levels after 14 weeks [18], beyond resistance training alone. It has recently been shown that low-dose creatine supplementation combined with protein supplementation and resistance training for 10 weeks increases lean tissue mass but not leg press strength [19]. Furthermore, there was a greater response in creatine combined with protein supplementation than creatine alone or placebo. Conversely, neither creatine supplementation, protein supplementation, or a combination of protein and creatine was shown to provide further benefit beyond 16 weeks of isotonic resistance training alone in elderly subjects [20].

In light of these controversial results, an animal model of hypertrophy (surgical ablation/compensatory hypertrophy) was chosen to compare the effects of creatine supplementation on hypertrophy in young and aging rats. Surgical ablation is an animal model involving surgical removal of a muscle, thereby overloading its synergistic muscles and causing them to hypertrophy in compensation [21–23]. The authors are aware of one other study that used this model to study creatine supplementation. Dangott et al. [24] showed no further hypertrophy in muscle fibers with creatine supplementation above compensatory hypertrophy alone. However, that study did not separate muscle into individual fiber types. Our study was designed to investigate whether creatine supplementation alone, surgical ablation alone, or a combination of creatine supplementation and surgical ablation would lead to hypertrophy in individual fiber types of the plantaris muscle. Furthermore, surgical ablation has been associated with increased expression of heat shock protein 70 (HSP70), which has been linked to myoprotection [25, 26] and anabolism [27, 28]. Therefore, the study examined whether these experimental treatments would alter HSP70 levels and whether hypertrophic and/or HSP70 responses would differ in young compared to older animals after these interventions. It was hypothesized that creatine supplementation would primarily increase CSA in fast fibers (IIA, IIB/D) and that this would be attenuated in aging muscle. We further hypothesized that HSP70 levels would be elevated in treatment groups undergoing surgical ablation but that creatine alone would not significantly alter HSP70 levels, regardless of age. 

## 2. Materials and Methods

### 2.1. Animals

Young (5 months at muscle excision, *N* = 27) and aging (24 months at muscle excision, *N* = 35) adult male Fisher 344 rats from National Institute on Aging rodent colony (Harlan Labs, Indianapolis, IN) were used. Animals were divided into eight groups: (1) young control (YC, *N* = 6); (2) young supplemented with creatine (YCr, *N* = 7); (3) young overloaded (YO, *N* = 7); (4) young overloaded and supplemented with creatine (YOCr, *N* = 7); (5) aging control (AC, *N* = 8); (6) aging supplemented with creatine (ACr, *N* = 9); (7) aging overloaded (AO, *N* = 9); (8) aging overloaded and supplemented with creatine (AOCr, *N* = 9). All rats were housed at the Ohio University Animal Facility. They were housed individually and provided with free access to rat chow and water. Temperature was maintained at 22°C ± 2°C, and conditions consisted of a 12-hour: 12-hour dark-light cycle. The experiments were approved by the IACUC of Ohio University.

### 2.2. Surgical Procedure

Animals were allowed 10 days of recovery from shipping and to become accustomed to their surroundings prior to experimentation. At that point, four experimental groups (YO, YOCr, AO, and AOCr) underwent surgical ablation surgery to induce overload by compensatory hypertrophy. Rats were anesthetized intraperitoneally by injection of either Veterinary Pentothol (*∼*0.07 mg/kg body weight) or a combination of Xylazine and Ketamine (0.01 g/kg body weight Xylazine; 0.01 g/kg body weight Ketamine). When there was no reflex response, an incision (*∼*2.5 cm) was made through the skin of the posterior compartment of the leg to expose the underlying muscle. The distal third of the gastrocnemius was removed bilaterally. Bilateral surgery ensured the animal would not favor either leg. The plantaris and the soleus muscles were left intact. Care was taken to ensure blood supply and innervation to remaining muscles were not compromised. The incision was sutured with 4.0 black braided silk and the stitches were approximately 0.3 cm apart, ensuring the incision was completely closed. After overnight recovery from the anesthetic, the animals were returned to their cages.

### 2.3. Creatine Supplementation

After returning to their cages, dietary creatine supplementation began for those in the creatine groups (YCr; YOCr; ACr; AOCr). Rats were given creatine monohydrate, 4.45 g/L (Sigma-Aldrich, St. Louis, MO) with a 5% dextrose solution (Sigma-Aldrich, St. Louis, MO) in the drinking water, *ad libitum*. Consumption of creatine solution was measured daily. The groups not receiving creatine were given 5% dextrose in the drinking water. The dextrose solution was chosen because creatine uptake is increased when combined with a carbohydrate solution [29]. Fluid levels for all rats were measured and replaced daily. Supplementation was continued for 4 weeks.

### 2.4. Muscle Extraction

Four weeks after the surgical ablation and/or beginning of creatine supplementation, the animals were killed, and the plantaris muscles were prepared for analysis. Samples were mounted for cross-sectioning in a mixture of tragacanth gum and optimal cutting temperature (OCT) cutting medium (Sakura Finetek Co., Torrence, CA) and frozen in methyl butane (Fisher Chemicals, Fair Lawn, NJ), cooled with liquid nitrogen. Samples were stored at −80°C until they were sectioned. Animals were subsequently euthanized by an anesthetic overdose, followed by heart removal. Samples were cryosectioned at −20°C to thicknesses of 8 *μ*m for ATPase analysis and 6 *μ*m for immunohistochemistry. Sections were placed onto cover slips coated with poly-L-lysine and stored at −40°C until the completion of histochemical analysis.

### 2.5. Immunohistochemistry

Serial sections were immunostained using primary antibodies against dystrophin (Sigma-Aldrich, St. Louis, MO) and laminin (Santa Cruz Biotechnology, Santa Cruz, CA); heat shock protein 70 (N27F3-4; Santa Cruz Biotechnology, Santa Cruz, CA); or myosin heavy chains (A4.591 and N2.261; Developmental Studies Hybridoma Bank, Iowa City, IA). The serial section stained for dystrophin and laminin were blocked with blocking serum, incubated with the primary antidystrophin antibody (4°C) overnight, washed with washing buffer, incubated in biotinylated secondary antibody (Vector, Burlingame, CA), washed, and labeled with the avidin-biotin complex (ABC) reagent conjugated with peroxidase. The sections were then incubated with antilaminin antibody (22°C) for 60 minutes and the same series of reactions repeated. The sections were then incubated in Nickel-enhanced diaminobenzidine (DAB; Pierce Chemical, Rockford, IL), counterstained with Delafield's hematoxylin and dehydrated in a graded alcohol series, before clearing and mounting. All other serial sections were incubated in primary antibody for one hour at 37°C, followed by the biotinylated secondary antibody, ABC reagent, dehydration and mounting as described above.

### 2.6. Fiber Typing

Sections were stained for mATPase activity, using previously described techniques [30]. In brief, serial sections were preincubated in a solution made to pH 4.15 or pH 4.45. These pH preincubations allowed Type I, Type IIA, and Type IIB/D fibers to be characterized. Myosin immunohistochemistry was performed on a subset of muscle samples to validate the mATPase-based fiber type delineation.

### 2.7. Cross-Sectional Area and Myonuclei

Cross-sectional area (CSA) was measured for each muscle fiber on a computer using a video captured microscope image of the cells stained with dystrophin and laminin and NIH Image software. Cross-sectional area was calculated for at least 300 fibers (from 3 random microscope frames *∼*0.56 mm^2^), or all the fibers in the sample. 

The myonuclei were counted at 1000X magnification and were identified due to their location inside the sarcolemma and the basal lamina. The total number per fiber type and total number per 100 fibers (%) were counted. Adjacent sections prepared for myofibrillar ATPase were matched with these fibers to characterize the fiber type and its properties.

### 2.8. Myonuclear Domain

Within a fiber type, the myonuclei/cytoplasm (N/C) ratio was determined to estimate myonuclear domain (mean CSA/mean myonuclei) as previously published [31, 32]. Myonuclear domain was compared between the 8 experimental conditions for a given fiber type.

### 2.9. Heat Shock Protein Quantification

**Figure 1:**
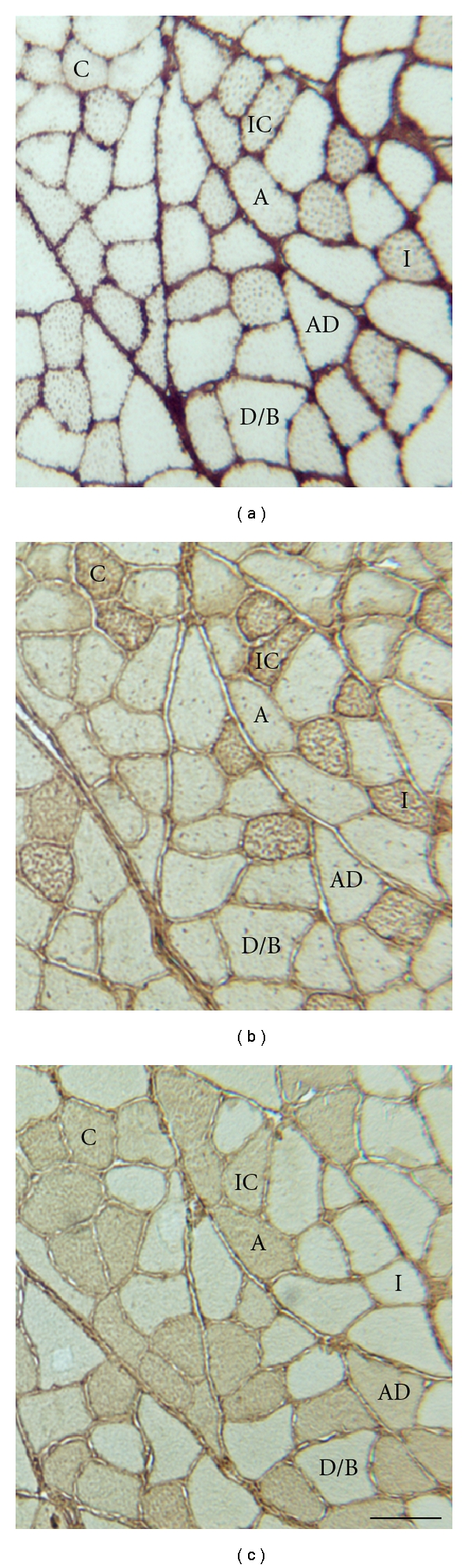
Immunohistochemical analysis of the plantaris. Micrographs representative of immunohistochemical assays for (a) HSP70, (b) MHC I, and (c) MHC II (Bar = 100 *μ*m).

Digital images of samples stained for HSP70 were acquired using video-capture microscopy (Figure 1). Light and diaphragm settings were held constant for all image acquisition. To assess HSP70 content, pixel density was determined on at least 50 fibers, of each major fiber type, per sample using NIH Image software. All pixel density figures were normalized to an acellular background on the coverslip of the sample.

### 2.10. Statistical Analysis

All statistical analyses were performed using Systat 11. A two-way analysis of variance (ANOVA) was used to compare the dependent variables (creatine and overload) across ages and muscle fiber types. Significance was set at *P* ≤ 0.05. Where significance was found for main effects, Fisher's least significant difference method was used for post hoc analysis. One-way ANOVA was used to compare creatine consumption, muscle mass, and body mass across ages. Student's-tests were used for animal and muscle mass analyses. Significance was set at *P* ≤ 0.05. Where significance was found for main effects, Fisher's least significant difference method was used for post-hoc analysis.

## 3. Results

In the interest of full disclosure, some data from the control groups (both young and aging) have been previously reported in Acta Physiologica [31]. However, no data from either age category within the treatment groups (Cr, O, or OCr) have been previously published, apart from conference abstracts. Furthermore, this manuscript contains new data from the control groups (e.g., HSP70). 

### 3.1. Fluid Consumption and Creatine Intake

Fluid intake is shown in Table 1. Older animals took in more creatine solution than young (*P* < 0.05). For the duration of the study, all animals had consistently more creatine intake than recommended for loading dose (0.3 g/kg body weight/day) and maintenance dose (0.03 g/kg body weight/day) as advised in the literature by Kreider and colleagues [33].

### 3.2. Body Mass and Muscle Mass

Body mass was significantly heavier in the aging rats, regardless of the treatment group (Table 2). The within-subject difference in body mass between pre- and posttreatment showed both aging groups that underwent surgical overload (AO and AOCr) lost mass. Compared to the young overloaded animals (YO and YOCr), these differences were significant (Table 2). In both the young and aging animals, the plantaris muscle of OCr group was heavier than control or creatine (young 30% heavier and older 18% heavier; *P* < 0.05; Table 3). The ratio of plantaris weight/body weight was also greater in the OCr group than control or creatine in both young and aging animals. In each experimental group, older muscles were heavier than young muscles in all groups (Table 3).

### 3.3. Young versus Aging Control Muscle

**Figure 2:**
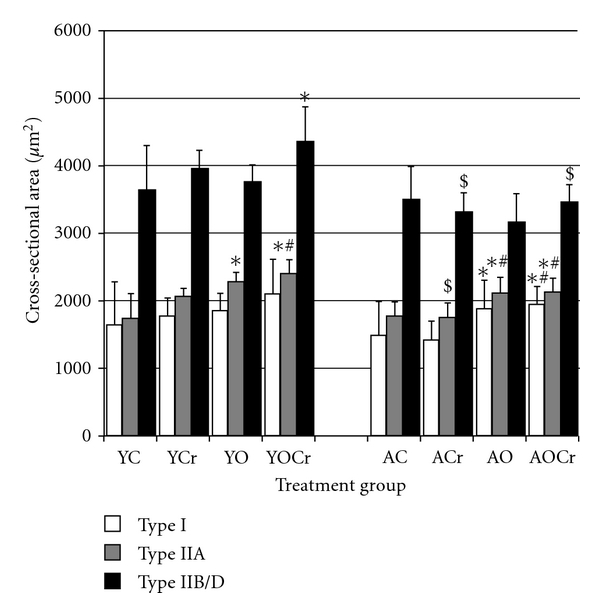
Cross-sectional area for plantaris muscle in young and aging animals for all treatment groups. Open bars are type I fibers, grey bars are type IIA fibers, and closed bars are IID/B fibers. Data are presented as mean ± SD. *Significantly different than control treatment, within age group (*P* < 0.05); ^#^significantly different than creatine group, within age group (*P* < 0.05); ^$^significantly different than same treatment in young rats (*P* < 0.05).

**Figure 3:**
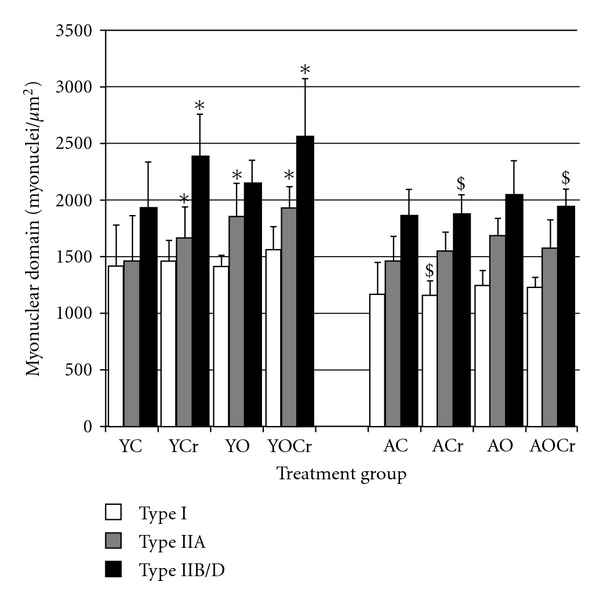
Myonuclear domain size for plantaris muscle in young and aging animals for all treatment groups. Open bars are type I fibers, grey bars are type IIA fibers, and closed bars are IID/B fibers. Data are presented as mean ± SD. *Significantly different than control treatment, within age group (*P* < 0.05); ^#^significantly different than creatine group, within age group (*P* < 0.05); ^$^significantly different than same treatment in young rats (*P* < 0.05). Units for myonuclear domain are myonuclei/um^2^.

**Figure 4:**
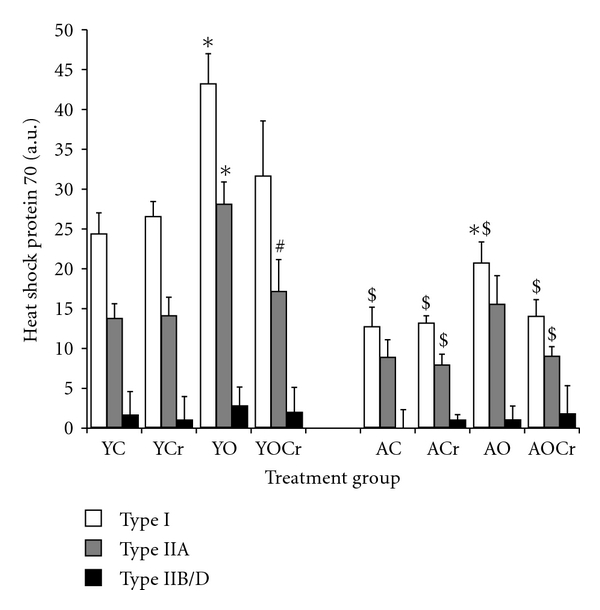
Heat shock protein for plantaris muscle in young and aging animals for all treatment groups. *indicates significantly different than control within age group (*P* < 0.05); ^#^Indicates significantly different than surgical overload treatment within age group (*P* < 0.05); ^$^significantly different than same treatment in young rats (*P* < 0.05).

The plantaris muscle was composed of predominantly fast fibers. Samples from aging animals had a higher percentage type I fibers and a lower percentage type IIA fibers than young animals (Table 4). The percentage of type IIB/D fibers was similar between young and aging animals. CSA data for type I, IIA, and IIB/D fibers are presented in Figure 2. There were no differences in CSA between young and older control animals for any fiber type. CSA for IIB/D was greater than I or IIA for all treatment groups (*P* < 0.05). Myonuclear domain size of type IIB/D fibers was significantly larger than I or IIA fibers within age groups of control animals (*P* < 0.05; Figure 3). There was no difference in myonuclear domain between type I and IIA fibers, within age groups. Type I and IIA fibers of young control animals had significantly higher HSP70 content than in those same fiber types of aging control animals (Figure 4). In the faster fibers of the plantaris, there was no significant difference in HSP70 content between young and aging animals of the control cohorts.

### 3.4. Creatine Supplementation

Creatine supplementation had no significant effect on fiber type composition. Fiber CSA did not significantly differ in animals receiving creatine compared to control rats, regardless of fiber type or age. However, trends toward significance were seen between YC and YCr treatment groups for CSA of type IIA (*P* = 0.057) and IIB/D (*P* = 0.087) fibers (2059.00 ± 115.28 *μ*m^2^ versus 1724.26 ± 380.61 *μ*m^2^, 28% difference and 3955.57 ± 264.98 *μ*m^2^ versus 3642.043 ± 648.06 *μ*m^2^, 17% difference, resp.; Figure 2). In aging rats, creatine supplementation did not significantly affect fiber CSA for any fiber type. The effects of creatine supplementation on myonuclear domain were both age and fiber type specific. In type I fibers of young animals, the myonuclear domain size was not different between creatine supplementation and control animals. In young animals, the myonuclear domain sizes of type IIA and IIB/D fibers were significantly larger in creatine supplemented rats than in control rats (IIA YCr = 1667.02 ± 274.34 *μ*m^2^ versus YC = 1463.63 ± 400.77 *μ*m^2^ and IIB/D YCr = 2386.83 ± 368.47 *μ*m^2^ versus YC = 1930.17 ± 405.09 *μ*m^2^; Figure 3). Creatine supplementation had no effect on myonuclear domain size in aging animals compared to control animals, regardless of fiber type. Myonuclear domain was significantly different between fiber types within age groups of creatine-supplemented animals (*P* < 0.05), except there was no difference between type I and IIA in YCr (*P* = 0.266). Creatine supplementation alone had no significant effects on HSP70 content for any age or fiber type.

### 3.5. Muscle Overload

In the overload group, young animals had significantly more type I fibers than control animals of a similar age (Table 4). There was no difference between the percentages of type I fibers in young and aging animals of the overload treatment. This was different from the other groups in which the older animals had higher percentages of type I fibers than the young animals (Table 4). Regardless of age, the percentage of type IIB/D fibers was lower in the overloaded group than in the control group. 

Surgical overload significantly increased the CSA of IIA fibers in the plantaris of young rats (35% difference, 2270.01 ± 142.84 *μ*m^2^ versus 1724.26 ± 380.61 *μ*m^2^, Figure 1). The CSAs of type I and IIB/D fibers from overloaded rats were larger than those fibers in control animals, but these data failed to reach significance due to large variability in the control animals (I 29%; 1841.68 ± 196.98 *μ*m^2^ versus 1635.95 ± 600.12 *μ*m^2^; IIB/D 12%; 3747.01 ± 263.40 *μ*m^2^ versus 3642.04 ± 648.06 *μ*m^2^; Figure 1). In the older animals, overload induced fiber hypertrophy of the slow-twitch fiber (21%; 1879.37 ± 212.47 *μ*m^2^ versus 1491.10 ± 299.40 *μ*m^2^) and IIA (16%; 2112.83 ± 226.32 *μ*m^2^ versus 1768.15 ± 209.31 *μ*m^2^) fiber types compared to the control group (Figure 2). However, CSA of type IIB/D fibers of aging animals were not significantly different between overloaded and control groups (Figure 2). 

Overload had very little effect on myonuclear domain. Only type IIA fibers of young, overloaded rats experienced a significant increase in myonuclear domain (1858.96 ± 287.91 *μ*m^2^ versus 1463.63 ± 400.77 *μ*m^2^; Figure 3). Overload did not cause any other significant changes in myonuclear domain size for any fiber type, irrespective of age (*P* > 0.05; Figure 3). Myonuclear domain size was smallest in type I fibers; type IIA and IIB/D had significantly larger domain sizes than type I in both young and older animals. Myonuclear domain sizes were larger in type IIA fibers than type I fibers of older animals compared to young animals (*P* < 0.05). Myonuclear domain was significantly different between fiber types within age groups of overloaded muscle.

Surgical overload resulted in significantly elevated HSP70 content, relative to control animals. This was true in type I and IIA fibers of YO and AO cohorts. However, overload did not affect the HSP 70 content of type IID/B, regardless of age group.

### 3.6. Overload and Creatine Supplementation

A combination of overload and creatine supplementation did not have the same influence on fiber type composition as overload alone. In young rats, the YOCr treatment group had a significantly lower percentage of type I fibers than the YO treatment. Furthermore, the percentage of type I in the YOCr group was similar to young controls. In young rats, the overloaded plantaris had a significantly higher percentage of type I fibers than overloaded plantaris from rats that also received creatine. However, similar to the control group, older animals in the AOCr group had a significantly higher percentage of type I, and a significantly lower percentage of type IIA fibers than young animals (Table 4). By contrast, overload alone did not result in significant changes to fiber type composition in aging animals. 

A combination of creatine and surgical overload produced significant hypertrophy in most muscle fibers. In young animals, the OCr treatment significantly increased fiber CSA in type IIA and IIB/D fibers, relative to young controls (IIA 28%, YOCr = 2401.51 ± 199.17 *μ*m^2^ versus YC = 1724.26 ± 380.61 *μ*m^2^; and IIB/D 20%, YOCr = 4352.28 ± 519.12 *μ*m^2^ versus YC = 3642.04 ± 648.06 *μ*m^2^; resp.). In older animals, only type I and IIA fibers of the OCr group were significantly larger than those fibers in the aging control animals (I 23%, AOCr = 1949.01 ± 180.77 *μ*m^2^ versus AC  = 1491.10 ± 299.40 *μ*m^2^; IIA17%, AOCr = 2125.66 ± 205.74 *μ*m^2^ versus AC  = 1768.15 ± 209.31 *μ*m^2^, resp.). Type IIB/D fibers of older animals in the OCr group did not change fiber sizes over controls (Figure 2).

The combined effects of creatine and overload differentially affected the myonuclear domains in the plantaris muscle of young and aging rats. For type IIA and IIB/D fibers, myonuclear domain was significantly larger in fibers of the YOCr group than in fibers from YC (IIA 24%, YOCr = 1934.58 ± 185.16 *μ*m^2^ versus YC = 1463.63 ± 400.77 *μ*m^2^; IIB/D 25%, YOCr = 2559.80 ± 510.02 *μ*m^2^ versus YC = 1930.17 ± 405.09 *μ*m^2^). In older rats, the myonuclear domain was not significantly affected by combined creatine and overload, regardless of the fiber type (*P* > 0.05). Myonuclear domain was significantly different between fiber types within age groups of animals in the overload plus creatine supplementation groups, except there was no difference between type I and IIA in YOCr (*P* = 0.102).

The combination of creatine and overload also resulted in significantly greater fiber CSA than creatine alone in fiber types I and IIA, regardless of age. For the CSA of type IIB/D fibers, there was a trend toward significance between YOCr and YCr groups (*P* = 0.051). This trend was not apparent in the aging animals. The CSA of type IIB/D fibers were significantly larger in YOCr than in YO animals, and there was a similar trend in the CSA of type I fibers (*P* = 0.057). In aging animals, there were no significant differences between the CSA of AOCr and AO for any fiber types. 

Type IIA fibers in YOCr animals had significantly lower HSP70 content than in YO. However, HSP70 content of IIA fibers of YOCr did not differ in type IIA fibers of YC or YCr cohorts. Creatine plus overload did not affect HSP70 expression in the aging group, regardless of fiber type.

## 4. Discussion

Research has shown that creatine supplementation, in combination with resistance training, can be beneficial in increasing lean body mass and strength (reviewed in [34]). Only a few studies have looked at creatine supplementation alone in skeletal muscle tissue. Fewer still have examined the fiber type-specific effects of creatine supplementation on CSA of aging muscle fibers. This study reports that the plantaris muscles of young and aging rats respond similarly to surgical overload. This is true for fiber type composition, cross-sectional area, and myonuclear domain size. Further, our data suggest that creatine supplementation alone had no significant effect on fiber CSA or HSP70 content, regardless of age or fiber type and the combination of overload and creatine also had minimal additional effect, relative to overload alone.

### 4.1. Creatine Supplementation

One specific aim of this research was to assess the efficacy of creatine supplementation in aging muscle. Few studies have examined the effects of creatine supplementation in the absence of exercise. Creatine supplementation without exercise did not enhance anabolic signals [35] or significantly alter fiber CSA [24]. Our data agree with these findings-creatine supplementation alone had no significant effect on fiber CSA in young or aging animals, regardless of the fiber type. *In vitro, *creatine has been shown to increase myosin heavy chain expression at both mRNA and protein levels [36], but our data did not agree with these findings. 

Creatine supplementation alone had no effect on CSA or myonuclear domain for any fiber type in the plantaris of aging animals. The authors are not aware of any previous research that investigated fiber-specific changes subsequent to creatine supplementation in aging muscle, so we propose the following possible explanations for our findings. Creatine supplementation significantly increases the myoprotective effects of carnosine and anserine in young, but not older animals [37]. Additionally, although creatine supplementation increased muscle phosphocreatine concentrations in both young and older adults, this response was significantly greater in the young adults [10]. Nonsupplementation studies also indicate that aging muscle is less capable of synthesizing or storing creatine. Compared to young skeletal muscle, aging muscle has fewer stores of PCr and Cr and a lower rate of PCr resynthesis [38].

### 4.2. Overload Surgery

A second specific aim of the current research was to examine the ability of aging animals to respond to a hypertrophic stimulus. Current results indicate that the slower fiber phenotypes of aging animals maintain their ability to hypertrophy in response to synergist ablation. These data also indicate that the overload-induced fast-to-slow fiber transition remains intact in older animals. These findings are in agreement with much of the literature related to compensatory hypertrophy of young muscle [23, 39]. Relatively few studies have investigated compensatory hypertrophy in older animals. The young and aging animals in this study responded similarly to the overload surgery, demonstrating that older muscle maintains the ability to hypertrophy. These findings are in contrast to a few studies that found a blunted response to overload in elderly animals [40–42]. These differences may be explained by the age difference in the “aging” group versus their elderly animals. Although the muscle fibers of the 24 month older rats did not exhibit age-associated atrophy, they did present other characteristics associated with aging, such as a slower fiber phenotype. In addition, the plantaris of the older groups had significantly higher muscle mass compared to the young groups. The increased muscle mass in the absence of fiber hypertrophy may reflect a reduction in muscle quality. Infiltrations of fat and noncontractile material are often noted in aging muscle [43, 44].

### 4.3. Overload and Creatine Supplementation

A third specific aim of this research was to examine the response of aging muscle to a combination of creatine and overload. Compared to the control group, the OCr treatment resulted in significant hypertrophy and significant increase in myonuclear domain for IIA and IIB/D fibers in the young plantaris muscle. In aging plantaris muscle, the combination of creatine and overload resulted in significant hypertrophy of types I and IIA fibers, but myonuclear domain sizes were not significantly different between control animals and animals in the OCr group for any fiber types. Compared to creatine alone, OCr groups had significantly larger CSA of type IIA fibers, regardless of age. 

The additional benefit of creatine in the OCr group over the O group was minimal. It is not immediately clear why the combination of creatine and overload did not result in significantly greater hypertrophy than overload alone. A combination of creatine supplementation and resistance training has previously been shown to result in larger fiber CSA [45–47]. However, the present findings are in agreement with R. E. Young, and J. C. Young [48], who demonstrated that creatine supplementation provided no additional hypertrophy beyond synergist ablation alone in soleus and extensor digitorum longus muscles. It is possible that the overload model is not appropriate for examining the effects of creatine on resistance exercise. The proposed benefit of creatine supplementation is to increase PCr stores, thereby increasing the capacity to regenerate ATP stores during short bursts of exercise [49]. In this scenario, the main benefit of creatine supplementation is to sustain high-intensity work for a longer duration [50]. In turn, the enhanced capacity to anaerobically train the muscle leads to increases in muscle mass and strength [7]. In the synergist ablation model, the overload imposed upon the muscle is permanent, and therefore cannot be considered anaerobic exercise. 

Creatine supplementation combined with overload surgery also seems to influence the shift in muscle phenotype to a slower phenotype. Interestingly, although both overloaded muscle and aging muscle demonstrated an increase in slow fibers, creatine supplemented animals of the aging group did not show that fast-to-slow shift in fiber types in either ACr or AOCr groups. This appears to show that the fiber type shift in aging animals can potentially be altered or reduced. 

There is a different response between young and older animals with regards to fiber type shift in response to overload. The young animals have an increase in the percentage of both type I and type IIA fibers after overload, which is similar to previous reports [23, 39]. However, in the aging overload, and the young and aging OCr groups, there is a shift from IIB/D to IIA, without a shift from IIA to I. Although the young overload group had the greatest fast-to-slow shift in fiber phenotype, this was not significantly different from the percent type I fibers in the aging animals with overload alone. The aging animals had already undergone an age-related fast-to-slow fiber shift, and the overload did not produce an additional phenotype shift. Creatine appears to blunt the overload-induced fast-to-slow fiber transition in the YOCr group. However, since creatine alone does not cause any significant shift in fiber type, we can only speculate that it must be an interaction between overload and creatine that negated the fast-to-slow shift seen in the YO group. In addition, the young OCr group had the greatest percent increases in CSA indicating the greatest response to the interventions.

### 4.4. Myonuclear Domain

A further specific aim of this project was to examine the age-specific effects of creatine and compensatory hypertrophy on myonuclear domain size. Creatine supplementation alone resulted in a significant increase in myonuclear domain of types IIA and IIB/D fibers in young animals whereas it had no effect on myonuclear domain size in aging animals. An increase in myonuclear domain implies that fiber size increased at a disproportionately higher rate than myonuclear addition. The results of this study indicate that creatine alone resulted in trends toward hypertrophy in young, but not aging, muscle fibers. Therefore, it is not surprising that the creatine-induced increase of myonuclear domain occurred only in the young animals. It is not clear why the myonuclear domain size of type I fibers did not significantly increase with creatine supplementation; however, fast-twitch fibers have relatively larger myonuclear domain sizes than slow-twitch fibers [51]. 

The concept of myonuclear domain implies that there is an optimal amount of genetic material to govern the structural proteins in that domain. It has been reported that if a fiber hypertrophies beyond 26%, myonuclei are added to maintain a constant myonuclear domain [52]. In young animals, type IIA fibers from the YCr, YO, and YOCr treatment groups were all hypertrophied more than 26%. Of these, the myonuclear domain size of the IIA fibers was also increased in all three treatments. It is possible that the fibers were in the process of increasing in size, and the myonuclei had not yet had a chance to proliferate.

### 4.5. Heat Shock Protein 70

It appears that HSP70 could assist in prevention of age-associated sarcopenia. Indeed, transgenic overexpression of HSP70 in aging rats was able to rescue damage-associated force deficits and speed recovery of the muscles [53]. Furthermore, both induced HSP70 and HSP70 overexpression attenuated unloading-induced muscle atrophy [28]. This atrophy-attenuating quality of HSP70 is due, at least in part, to the repression of the FOXO3a-induced transcription of atrogin-1 [27]. 

Our data demonstrate a significant decrease in HSP70 content in type I and IIA fibers of aging rats, relative to young rats. These findings partially agree with Naito and colleagues [54], who found an insignificant decrease in HSP70 content in the plantaris muscle of aging rats, relative to young rats. In that study, HSP70 content was measured in muscle homogenates, not individual fiber types. Given the high percentage of type IID/B fibers in the plantaris and the low HSP70 content in type IID/B fibers, it is reasonable to conclude that homogenizing the muscle, as done in [54], diluted any age-related differences that may have occurred in HSP70 content. Conversely, Chung and Ng [55] demonstrated a significant increase in HSP70 content in aging rats. It is not possible to directly compare the two studies due to differences in muscle (plantaris versus gastrocnemius), technique (histochemistry versus Western blot), and ages used (5 m–24 m versus 16 m–29 m). 

Our data indicate that creatine supplementation alone has no effect on HSP70 content. Further, our data demonstrate a creatine-induced attenuation to the overload-associated increase in HSP70 content, as evidenced by the lack of significance between C and OCr groups. To our knowledge, these data are the first to examine the effects of creatine supplementation on HSP70 content. However, a link has been established between energy availability and HSP70 expression. Exercise resulted in increased HSP70 expression of glycogen-depleted skeletal muscle but not in glycogen-containing control muscle [56]. It has also been demonstrated that ADP acts as a cofactor in HSP70-peptide binding interactions, and binding of HSP70 is directly correlated to the ADP/ATP ratio [57]. A function of creatine supplementation is restoring ATP [49]. Therefore, the attenuation of HSP70 content in OCr groups, relative to overload alone, may be explained by the ability of creatine to maintain ATP levels.

In the current study, the aging muscle shows an inability to respond to creatine and (in comparison with the young animals) a blunted response to overload. If creatine acts directly on satellite cells, as has been shown in cell culture [58], then the age-associated loss of responsiveness to creatine may be a result of dysfunctional satellite cells. In young individuals, creatine increased the quantity of both satellite cells and myonuclei in young males, contributing to enhanced growth [59]. 

Many age-related studies that have used a rat model have documented an increase in body mass throughout the animal's lifespan. Fisher 344 rats were selected for this study, because the body mass of this strain plateaus. 24 months of age was selected for the aging group, because the NIH survival curves indicate a 50% survival for Fisher 344 rats at this age. Furthermore, 24-month-old Fisher 344 rats are analogous to 80-year-old humans [60]. Age-associated changes in fiber type and CSA often accompany animals in this age range [42, 61]. While the skeletal muscle of the aging animals in this study did not exhibit some of the characteristics commonly associated with sarcopenia, the aim of this study was to determine whether the C/N characteristics and responsiveness to overload and creatine change during the aging process. The data demonstrate that these age-associated changes did occur, despite the absence of age-associated declines of fast fiber types and fiber CSA.

### 4.6. Limitations

Interpretation of the present data is complicated by a few limitations of the study. First, creatine uptake by the muscle was not measured. Therefore, it is not clear whether age-specific results were due to the inability of aging muscle to take in creatine or to respond to the creatine. Previous research has shown that creatine transporters are not influenced by aging or activity [62]. Secondly, the compensatory hypertrophy model is an involuntary model of extreme overload. Though it is a valid, common technique for inducing hypertrophy in murine models, humans are incapable of voluntarily exercising at this level of intensity. Furthermore, creatine is believed to confer its effect on muscle by replenishing anaerobic energy stores, thereby allowing users to perform an additional repetition of resistance exercise. As such, creatine supplementation may result in greater gains if the training intensity can be increased throughout the training period. For example, significant increases in fat-free mass and strength were seen when creatine supplementation was coupled with resistance training, modified to maintain maximal effort [18]. It should be noted that in the Brose et al. [18] study, fiber CSA was similar between creatine- and placebo-supplemented groups. Nevertheless, compensatory hypertrophy is a form of permanent overload that cannot be modified incrementally and is a limitation of the study. Lastly, this study did not quantify protein synthesis or degradation. The ratio of protein synthesis and degradation is known to change with aging [1]. Creatine supplementation has been linked to protein synthesis [47]; therefore, measuring these indices may have enhanced the study.

### 4.7. Summary

Given the prevalence of age-associated sarcopenia, the search for a preventative supplement is of vital importance. The data presented here indicate that creatine supplementation alone does not affect fiber hypertrophy in aging muscle. Furthermore, this study found that a combination of creatine and overload was no more effective than overload alone on hypertrophy. In fact, the creatine supplementation appeared to result in a small, but significant, attenuation of overload-induced HSP70 expression.

## Figures and Tables

**Table 1:** Fluid consumption and creatine intake for young and aging rats.

	Young		Aging	
	Fluid intake (mL/day)	Creatine intake (g/kg body wt)	Fluid intake (mL/day)	Creatine intake (g/kg body wt)
Control	29 ± 6 mL/day		36 ± 4 mL/day	
Creatine	30 ± 6 mL/day	0.41 ± 0.07 g/kg	40 ± 4 mL/day	0.47 ± 0.12 g/kg
Overload	28 ± 4 mL/day		43 ± 10 mL/day	
Overload + Creatine	28 ± 2 mL/day	0.42 ± 0.03 g/kg	50 ± 9 mL/day^a^	0.58 ± 0.10 g/kg^b^

Data presented as Mean ± SD. ^a^ *P* < 0.05, greater than control group; ^b^ *P* < 0.05, old greater than young.

**Table 2:** Pre- and posttreatment weight of young and aging animals.

	Young			Aging		
	Weight (Pre; g)	Weight (Post; g)	Change in Weight (Post-Pre; g)	Weight (Pre; g)	Weight (Post; g)	Change in Weight (Post-Pre; g)
Control	273.3 ± 27.8	296.0 ± 31.7	+22.8 ± 18.6	393.0 ± 25.8	416.8 ± 27.5^d^	+23.8 ± 9.1
Creatine	285.4 ± 25.6	324.9 ± 25.4	+39.5 ± 14.4	373.8 ± 15.4	408.9 ± 19.7^d^	+35.2 ± 8.3^a^
Overload	278.9 ± 12.7	302.6 ± 18.2	+23.8 ± 7.3	411.3 ± 21.5	388.8 ± 23. 2^d^	−22.6 ± 12.2^bc^
Overload + creatine	272.8 ± 12.4	302.3 ± 11.6	+29.5 ± 7.6	394.2 ± 15.0	384.1 ± 18.0^d^	−10.1 ± 9.5^c^

Data presented as Mean ± SD. ^a^ *P* < 0.001, greater than overload and OCr groups; ^b^ *P* < 0.05, less than OCr group; ^c^ *P* < 0.001, less body weight increase than young; ^d^ *P* < 0.05, heavier than same treatment in young.

**Table 3:** Plantaris muscle weight and muscle weight/body weight ratio for young and aging animals.

	Young		Aging	
	Plantaris Wt (g)	Ratio plantaris	Plantaris Wt (g)	Ratio plantaris
		Wt/Body Wt		Wt/Body Wt
Control	0.236 ± 0.030	0.080 ± 0.011	0.319 ± 0.040^c^	0.077 ± 0.009
Creatine	0.255 ± 0.030	0.080 ± 0.009	0.328 ± 0.037^c^	0.080 ± 0.009
Overload	0.276 ± 0.050	0.091 ± 0.015	0.358 ± 0.040^c^	0.092 ± 0.010^b^
Overload + creatine	0.308 ± 0.019^a^	0.102 ± 0.003^b^	0.375 ± 0.037^ac^	0.098 ± 0.008^b^

Data presented as Mean ± SD. ^a^ *P* < 0.05, heavier than control or creatine groups; ^b^ *P* < 0.05, higher ratio than control or creatine groups; ^c^ *P* < 0.05, heavier than same treatment in young.

**Table 4:** Percent fiber type of plantaris muscle for young and aging animals.

	Young			Aging		
	Type I %	Type IIA %	Type IIB/D %	Type I %	Type IIA %	Type IIB/D %
Control	8 ± 2^e^	19 ± 3	74 ± 4	14 ± 3^a^	13 ± 4^a^	73 ± 2
Creatine	6 ± 4^e^	22 ± 4^f^	71 ± 6^d^	13 ± 7^a,d^	11 ± 3^a,d^	77 ± 7
Overload	19 ± 7^c^	20 ± 5	62 ± 5^b,g^	18 ± 7	18 ± 6	64 ± 9^b,h^
Overload + creatine	10 ± 4^d^	24 ± 7	66 ± 9^b^	17 ± 5^a^	16 ± 5^a,g^	67 ± 5^g^

Data presented as Mean ± SD. ^a^aging significantly different than young within treatment *P* < 0.05; ^b^significantly different than control within age group *P* < 0.05; ^c^significantly different than control within age group *P* < 0.001; ^d^significantly different than overload within age group *P* < 0.05; ^e^significantly different than overload within age group *P* < 0.001; ^f^young significantly different than old within treatment *P* < 0.001; ^g^significantly different than creatine within age group *P* < 0.05; ^h^significantly different than creatine within age group *P* < 0.001.

## References

[1] Ryall JG, Schertzer JD, Lynch GS (2008). Cellular and molecular mechanisms underlying age-related skeletal muscle wasting and weakness. *Biogerontology*.

[2] Balsom PD, Soderlund K, Sjodin B, Ekblom B (1995). Skeletal muscle metabolism during short duration high-intensity exercise: influence of creatine supplementation. *Acta Physiologica Scandinavica*.

[3] Earnest CP, Snell PG, Rodriguez R, Almada AL, Mitchell TL (1995). The effect of creatine monohydrate ingestion on anaerobic power indices, muscular strength and body composition. *Acta Physiologica Scandinavica*.

[4] Rawson ES, Volek JS (2003). Effects of creatine supplementation and resistance training on muscle strength and weightlifting performance. *Journal of Strength and Conditioning Research*.

[5] Wallimann T, Wyss M, Brdiczka D, Nicolay K, Eppenberger HM (1992). Intracellular compartmentation, structure and function of creatine kinase isoenzymes in tissues with high and fluctuating energy demands: the ‘phosphocreatine circuit’ for cellular energy homeostasis. *Biochemical Journal*.

[6] Greenhaff PL, Bodin K, Soderlund K, Hultman E (1994). Effect of oral creatine supplementation on skeletal muscle phosphocreatine resynthesis. *American Journal of Physiology*.

[7] Kraemer WJ, Volek JS (1999). Creatine supplementation: its role in human performance. *Clinics in Sports Medicine*.

[8] Paddon-Jones D, Rasmussen BB (2009). Dietary protein recommendations and the prevention of sarcopenia. *Current Opinion in Clinical Nutrition and Metabolic Care*.

[9] Tarnopolsky MA (2000). Potential benefits of creatine monohydrate supplementation in the elderly. *Current Opinion in Clinical Nutrition and Metabolic Care*.

[10] Rawson ES, Clarkson PM, Price TB, Miles MP (2002). Differential response of muscle phosphocreatine to creatine supplementation in young and old subjects. *Acta Physiologica Scandinavica*.

[11] Rawson ES, Clarkson PM (2000). Acute creatine supplementation in older men. *International Journal of Sports Medicine*.

[12] Jakobi JM, Rice CL, Curtin SV, Marsh GD (2001). Neuromuscular properties and fatigue in older men following acute creatine supplementation. *European Journal of Applied Physiology*.

[13] Wiroth JB, Bermon S, Andreï S, Dalloz E, Hébuterne X, Dolisi C (2001). Effects of oral creatine supplementation on maximal pedalling performance in older adults. *European Journal of Applied Physiology*.

[14] Gotshalk LA, Volek JS, Staron RS, Denegar CR, Hagerman FC, Kraemer WJ (2002). Creatine supplementation improves muscular performance in older men. *Medicine and Science in Sports and Exercise*.

[15] Gotshalk LA, Kraemer WJ, Mendonca MAG (2008). Creatine supplementation improves muscular performance in older women. *European Journal of Applied Physiology*.

[16] Bermon S, Venembre P, Sachet C, Valour S, Dolisi C (1998). Effects of creatine monohydrate ingestion in sedentary and weight-trained older adults. *Acta Physiologica Scandinavica*.

[17] Chrusch MJ, Chilibeck PD, Chad KE, Davison KS, Burke DG (2001). Creatine supplementation combined with resistance training in older men. *Medicine and Science in Sports and Exercise*.

[18] Brose A, Parise G, Tarnopolsky MA (2003). Creatine supplementation enhances isometric strength and body composition improvements following strength exercise training in older adults. *The Journals of Gerontology: Series A*.

[19] Candow DG, Little JP, Chilibeck PD (2008). Low-dose creatine combined with protein during resistance training in older men. *Medicine and Science in Sports and Exercise*.

[20] Carter JM, Bemben DA, Knehans AW, Bemben MG, Witten MS (2005). Does nutritional supplementation influence adaptability of muscle to resistance training in men aged 48 to 72 years. *Journal of Geriatric Physical Therapy*.

[21] Baldwin KM, Valdez V, Herrick RE (1982). Biochemical properties of overloaded fast-twitch skeletal muscle. *Journal of Applied Physiology Respiratory Environmental and Exercise Physiology*.

[22] Goldberg AL, Etlinger JD, Goldspink DF, Jablecki C (1975). Mechanism of work induced hypertrophy of skeletal muscle. *Medicine and Science in Sports and Exercise*.

[23] Roy RR, Meadows ID, Baldwin KM, Edgerton VR (1982). Functional significance of compensatory overloaded rat fast muscle. *Journal of Applied Physiology Respiratory Environmental and Exercise Physiology*.

[24] Dangott B, Schultz E, Mozdziak PE (2000). Dietary creatine monohydrate supplementation increases satellite cell mitotic activity during compensatory hypertrophy. *International Journal of Sports Medicine*.

[25] Koh TJ (2002). Do small heat shock proteins protect skeletal muscle from injury?. *Exercise and Sport Sciences Reviews*.

[26] Srikakulam R, Winkelmann DA (2004). Chaperone-mediated folding and assembly of myosin in striated muscle. *Journal of Cell Science*.

[27] Senf SM, Dodd SL, Judge AR (2010). FOXO signaling is required for disuse muscle atrophy and is directly regulated by Hsp70. *American Journal of Physiology*.

[28] Takeda I, Fujino H, Murakami S, Kondo H, Nagatomo F, Ishihara A (2009). Thermal preconditioning prevents fiber type transformation of the unloading induced-atrophied muscle in rats. *Journal of Muscle Research and Cell Motility*.

[29] Green AL, Hultman E, Macdonald IA, Sewell DA, Greenhaff PL (1996). Carbohydrate ingestion augments skeletal muscle creatine accumulation during creatine supplementation in humans. *American Journal of Physiology*.

[30] Brooke MH, Kaiser KK (1970). Three “myosin adenosine triphosphatase” systems: the nature of their pH lability and sulfhydryl dependence. *Journal of Histochemistry and Cytochemistry*.

[31] Brooks NE, Schuenke MD, Hikida RS (2009). Ageing influences myonuclear domain size differently in fast and slow skeletal muscle of rats. *Acta Physiologica*.

[32] Hikida RS, Staron RS, Hagerman FC (2000). Effects of high-intensity resistance training on untrained older men. II. Muscle fiber characteristics and nucleo-cytoplasmic relationships. *The Journals of Gerontology: Series A*.

[33] Kreider RB, Ferreira M, Wilson M (1998). Effects of creatine supplementation on body composition, strength, and sprint performance. *Medicine and Science in Sports and Exercise*.

[34] Paddon-Jones D, Børsheim E, Wolfe RR (2004). Potential ergogenic effects of arginine and creatine supplementation. *Journal of Nutrition*.

[35] Deldicque L, Atherton P, Patel R (2008). Effects of resistance exercise with and without creatine supplementation on gene expression and cell signaling in human skeletal muscle. *Journal of Applied Physiology*.

[36] Ingwall JS, Morales MF, Stockdale FE (1972). Creatine and the control of myosin synthesis in differentiating skeletal muscle. *Proceedings of the National Academy of Sciences of the United States of America*.

[37] Derave W, Jones G, Hespel P, Harris RC (2008). Creatine supplementation augments skeletal muscle carnosine content in senescence-accelerated mice (SAMP8). *Rejuvenation Research*.

[38] Moller P, Bergstrom J, Furst P, Hellstrom K (1980). Effect of aging on energy-rich phosphagens in human skeletal muscles. *Clinical Science*.

[39] Roy RR, Baldwin KM, Martin TP (1985). Biochemical and physiological changes in overloaded rat fast- and slow-twitch ankle extensors. *Journal of Applied Physiology*.

[40] Alway SE, Degens H, Krishnamurthy G, Smith CA (2002). Potential role for Id myogenic repressors in apoptosis and attenuation of hypertrophy in muscles of aged rats. *American Journal of Physiology*.

[41] Blough ER, Linderman JK (2000). Lack of skeletal muscle hypertrophy in very aged male Fischer 344 x Brown Norway rats. *Journal of Applied Physiology*.

[42] Degens H, Alway SE (2003). Skeletal muscle function and hypertrophy are diminished in old age. *Muscle and Nerve*.

[43] Conley KE, Cress ME, Jubrias SA, Esselman PC, Odderson IR (1995). From muscle properties to human performance, using magnetic resonance. *The Journals of Gerontology: Series A*.

[44] Cree MG, Newcomer BR, Katsanos CS (2004). Intramuscular and liver triglycerides are increased in the elderly. *Journal of Clinical Endocrinology and Metabolism*.

[45] Volek JS, Ratamess NA, Rubin MR (2004). The effects of creatine supplementation on muscular performance and body composition responses to short-term resistance training overreaching. *European Journal of Applied Physiology*.

[46] Volek JS, Duncan ND, Mazzetti SA (1999). Performance and muscle fiber adaptations to creatine supplementation and heavy resistance training. *Medicine and Science in Sports and Exercise*.

[47] Willoughby DS, Rosene J (2001). Effects of oral creatine and resistance training on myosin heavy chain expression. *Medicine and Science in Sports and Exercise*.

[48] Young RE, Young JC (2007). The effect of creatine supplementation on mass and performance of rat skeletal muscle. *Life Sciences*.

[49] Brault JJ, Towse TF, Slade JM, Meyer RA (2007). Parallel increases in phosphocreatine and total creatine in human vastus lateralis muscle during creatine supplementation. *International Journal of Sport Nutrition and Exercise Metabolism*.

[50] McBride TA, Gregory MA (2002). Effect of creatine supplementation during high resistance training on mass, strength, and fatigue resistance in rat skeletal muscle. *Journal of Strength and Conditioning Research*.

[51] Tseng BS, Kasper CE, Edgerton VR (1994). Cytoplasm-to-myonucleus ratios and succinate dehydrogenase activities in adult rat slow and fast muscle fibers. *Cell and Tissue Research*.

[52] Kadi F, Schjerling P, Andersen LL (2004). The effects of heavy resistance training and detraining on satellite cells in human skeletal muscles. *Journal of Physiology*.

[53] McArdle A, Dillmann WH, Mestril R, Faulkner JA, Jackson MJ (2004). Overexpression of HSP70 in mouse skeletal muscle protects against muscle damage and age-related muscle dysfunction. *The FASEB Journal*.

[54] Naito H, Powers SK, Demirel HA, Aoki J (2001). Exercise training increases heat shock protein in skeletal muscles of old rats. *Medicine and Science in Sports and Exercise*.

[55] Chung L, Ng YC (2006). Age-related alterations in expression of apoptosis regulatory proteins and heat shock proteins in rat skeletal muscle. *Biochimica et Biophysica Acta*.

[56] Febbraio MA, Ott P, Nielsen HB (2002). Exercise induces hepatosplanchnic release of heat shock protein 72 in humans. *Journal of Physiology*.

[57] Glick BS (1995). Can Hsp70 proteins act as force-generating motors?. *Cell*.

[58] Vierck JL, Icenoggle DL, Bucci L, Dodson MV (2003). The effects of ergogenic compounds on myogenic satellite cells. *Medicine and Science in Sports and Exercise*.

[59] Olsen S, Aagaard P, Kadi F (2006). Creatine supplementation augments the increase in satellite cell and myonuclei number in human skeletal muscle induced by strength training. *Journal of Physiology*.

[60] Rice KM, Linderman JK, Kinnard RS, Blough ER (2005). The Fischer 344/NNiaHSd X Brown Norway/BiNia is a better model of sarcopenia than the Fischer 344/NNiaHSd: a comparative analysis of muscle mass and contractile properties in aging male rat models. *Biogerontology*.

[61] Lushaj EB, Johnson JK, McKenzie D, Aiken JM (2008). Sarcopenia accelerates at advanced ages in Fisher 344xBrown Norway rats. *The Journals of Gerontology: Series A*.

[62] Tarnopolsky M, Parise G, Fu MH (2003). Acute and moderate-term creatine monohydrate supplementation does not affect creatine transporter mRNA or protein content in either young of elderly humans. *Molecular and Cellular Biochemistry*.

